# Serial S100B Sampling Detects Intracranial Lesion Development in Patients on Extracorporeal Membrane Oxygenation

**DOI:** 10.3389/fneur.2019.00512

**Published:** 2019-05-16

**Authors:** Alexander Fletcher-Sandersjöö, Caroline Lindblad, Eric Peter Thelin, Jiri Bartek Jr., Marko Sallisalmi, Adrian Elmi-Terander, Mikael Svensson, Bo-Michael Bellander, Lars Mikael Broman

**Affiliations:** ^1^Department of Neurosurgery, Karolinska University Hospital, Stockholm, Sweden; ^2^Department of Clinical Neuroscience, Karolinska Institutet, Stockholm, Sweden; ^3^Division of Neurosurgery, Department of Clinical Neurosciences, University of Cambridge, Cambridge, United Kingdom; ^4^Department of Neurosurgery, Copenhagen University Hospital Rigshospitalet, Copenhagen, Denmark; ^5^Department of Medicine, Karolinska Institutet, Stockholm, Sweden; ^6^ECMO Center Karolinska, Karolinska University Hospital, Stockholm, Sweden; ^7^Department of Physiology and Pharmacology, Karolinska Institutet, Stockholm, Sweden

**Keywords:** S100B, extracorporeal membrane oxygenation, ECMO, intracranial hemorrhage, ischemic stroke, brain injury, intracranial lesion

## Abstract

**Introduction:** Intracranial lesion development is a recognized complication in adults treated with extracorporeal membrane oxygenation (ECMO) and is associated with increased mortality. As neurological assessment during ECMO treatment remains challenging, protein biomarkers of cerebral injury could provide an opportunity to detect intracranial lesion development at an early stage. The aim of this study was to determine if serially sampled S100B could be used to detect intracranial lesion development during ECMO treatment.

**Methods:** We conducted an observational cohort study of all patients treated with ECMO at ECMO Center Karolinska (Karolinska University Hospital, Stockholm, Sweden) between January and August 2018, excluding patients who did not undergo a computerized tomography scan (CT) during treatment. S100B was prospectively collected at hospital admission and then once daily. The primary end-point was any type of CT verified intracranial lesion. Receiver operating characteristics (ROC) curves and Cox proportional hazards models were employed.

**Results:** Twenty-nine patients were included, of which 15 (52%) developed an intracranial lesion and exhibited higher levels of S100B overall. S100B had a robust association with intracranial lesion development, especially during the first 200 hours following admission. The best area-under-curve (AUC) to predict intracranial lesion development was 40 and 140 hours following ECMO initiation, were a S100B level of 0.69μg/L had an AUC of 0.81 (0.628-0.997). S100B levels were markedly increased following the development of intracranial hemorrhage.

**Conclusions:** Serial serum S100B samples in ECMO patients were both significantly elevated and had an increasing trajectory in patients developing intracranial lesions. Larger prospective trials are warranted to validate these findings and to ascertain their clinical utility.

## Introduction

Extracorporeal membrane oxygenation (ECMO) is recognized as an important method of treatment for patients suffering from severe reversible refractory respiratory and/or circulatory failure (1–3). However, in addition to the critical condition of the patients accepted for treatment, ECMO itself is associated with significant morbidity and mortality (4). Neurological complications, including stroke and intracranial hemorrhage (ICH), are common and remain leading causes of ECMO-associated death worldwide (5–8). Of these, ICH, with a reported incidence of 1.8–21% and mortality rate of 32–100%, has a particularly poor prognosis (7).

Comprehensive neurological assessment during ECMO treatment is challenging, since patients are often deeply sedated. Moreover, invasive neuromonitoring in ECMO patients, including placement of intracranial pressure devices or external ventricular drains, is associated with a high risk of bleeding complications and death (9). Instead, non-invasive neurological monitoring could provide an opportunity to detect intracranial lesion development at an earlier stage, including protein biomarkers of cerebral injury (10), cerebral near infrared spectroscopy (NIRS) (11) and transcranial doppler (TCD) (12).

The most studied biomarker of cerebral injury is S100B, a protein predominantly expressed in perivascular astrocytes (13). Serum levels of S100B have been strongly associated with escalating brain injury severity in traumatic brain injury (TBI) cohorts (14), as well as with the progression of intracranial lesions (15). Serial sampling of S100B can also be used to detect cerebral deterioration in patients with TBI and subarachnoid hemorrhage, where it has been shown to assist in clinical decision making (16–18). However, the clinical utility of S100B in ECMO populations warrants further studies.

The aim of this study was to determine how serial S100B sampling can be used to detect intracranial lesion development during ECMO treatment.

## Methods

### Study Design

This was a retrospective observational cohort study, of prospectively collected S100B samples, including all patients treated with ECMO at ECMO Center Karolinska, Karolinska University Hospital (Stockholm, Sweden) between January 1st and August 10th 2018. S100B was collected and analyzed at hospital admission and daily during treatment. The primary endpoint was any type of computed tomography (CT) verified intracranial lesion, thus patients who did not undergo a CT scan were excluded. The intracranial lesions of primary interest were ischemia and ICH. ICH was defined as an intraparenchymal hemorrhage, subarachnoid hemorrhage or subdural hemorrhage. The study was approved by the Regional Ethical Review Board in Stockholm, Sweden (#2018/830-31).

### Patient Management

In most cases, ECMO was commenced at the referring hospital and the patient was then transferred to the ECMO ICU at the Karolinska University Hospital (19, 20). Anticoagulation was achieved by continuous intravenous infusion of unfractionated heparin targeting an APTT of 1.5–2 times the mean normal value, which was monitored at least three times daily. During treatment, a bedside ECMO specialist nurse regularly performed neurological checks, which included brainstem reflexes and pupillary examinations. While S100B was sampled, no study specific interpretations of the results were performed to trigger any specific management or diagnostics (CT scanning).

### S100B Sampling

Serum S100B was prospectively collected at hospital admission (venous) and then once daily at 06:00 AM (arterial). Samples were immediately sent to and analyzed at the Department of Clinical Chemistry, Karolinska University Hospital, Stockholm, Sweden using an automatic electrochemiluminescence immunoassay (Cobas; Roche Diagnostics, Basel, Switzerland). The measurement range for the assay is 0.005–39 μg/L.

### Variables

Medical history and clinical charts were retrospectively reviewed, and the following data were collected: age, age group (neonatal, pediatric or adult), sex, ECMO indication, ECMO mode, time of ECMO initiation and termination, S100B, time of CT scan and ICU mortality. ECMO indications were separated into organ system-specific categories (Supplementary Table 1). For all longitudinal calculations, time was defined as “time since the start of ECMO.” If S100B samples were taken before ECMO initiation, that time was considered <0. Neonatal patients were defined as ≤28 days of age, and only patients with a gestational age >34 weeks were considered for ECMO treatment. No adjustment was made with regards to gestational age.

### Statistical Analysis

Continuous variables were presented as mean ± standard deviation (SD) if normally distributed and otherwise median (interquartile range). Categorical variables were presented as count (%). The statistical software program R, with the graphical interface Rstudio® (21), was used in all calculations. The raw data is available as a supplementary file (Supplementary Dataset 1).

### Missing Data and Variable Assumptions

Missing values in the complete data set were examined graphically (Supplementary Figure 1) (22). The ECMO termination hour was the first minute of the day that ECMO treatment was terminated. For patients who had undergone a CT scan following a S100B measurement, the last observed S100B value preceding the CT scan was considered to be the S100B value at the time of the CT measurement [a last-observation-carried-forward approach (23)]. All patients were assumed *not* to have suffered any intracranial events preceding ECMO admittance. Between radiologic measurements, we estimated the outcome value (primary/secondary endpoints) through a last-observation-carried-forward approach.

### Inferential Analysis

In order to study the sensitivity and specificity of S100B to detect intracranial lesions, receiver operating characteristics (ROC) curves were used with “time since ECMO initiation” re-categorized using defined time intervals (Supplementary Table 2). If the same patient had multiple S100B measurements during these defined intervals, the mean S100B was used. Subsequently, a ROC curve was generated for each time interval, and the area under the ROC curve (AUC) with 95% confidence interval (CI) was calculated using the R package pROC (24). For the ROC curve with the largest AUC, the optimal S100B threshold was calculated using the Youden method (25), which is a common way of summarizing the performance of ROC curves. Among the generated ROC curve thresholds a bootstrap technique of 2000 stratified replicates was used to generate 95% confidence intervals for sensitivity and specificity for the “best threshold” also using the pROC package in R. Timing of optimal S100B sampling to detect intracranial lesions was assessed using a sliding window approach, similar to previous work from our group (14, 15).

In order to evaluate the risk for development of an intracranial lesion over time, we used Cox proportional hazards model (26), with S100B measurements as a time-varying covariate (27). For analysis, we employed the survival package in R (28, 29). Data was presented as hazard ratio (HR) and 95% CI. Model assumptions were tested using the R packages survminer (30), tidyverse (31), and survival (29). Specifically, we assessed log-linearity and the proportional hazards assumption. For the proportional hazards assumption, we calculated and subsequently plotted the so called Schoenfeld residuals (i.e., the residuals used to determine the model's time-independence) (Supplementary Figures 2, 3). Overall model significance was checked using Robust Log-Rank test and Wald Test synchronously, since these do *not* assume independence among observations obtained from the same patient (29). Throughout all these analyses, base 10-logarithmic S100B was used to meet model assumptions. Since there were few patients in the material, we abstained from using a multivariable approach.

### Subgroup Analysis

Timing of presumptive CT scans was generally conducted at the discretion of the attending physician. However, previous work with serial sampling of S100B has shown an important correlation between secondary increases (“peaks”) of S100B and occurrence of new CT-verifiable lesions (17). In order to account for this, we chose a subset of patients that had undergone CT scans *after* S100B peaks, and these patients were analyzed using the same Cox proportional hazards model as described above.

## Results

### Demographics

**Figure 1:**
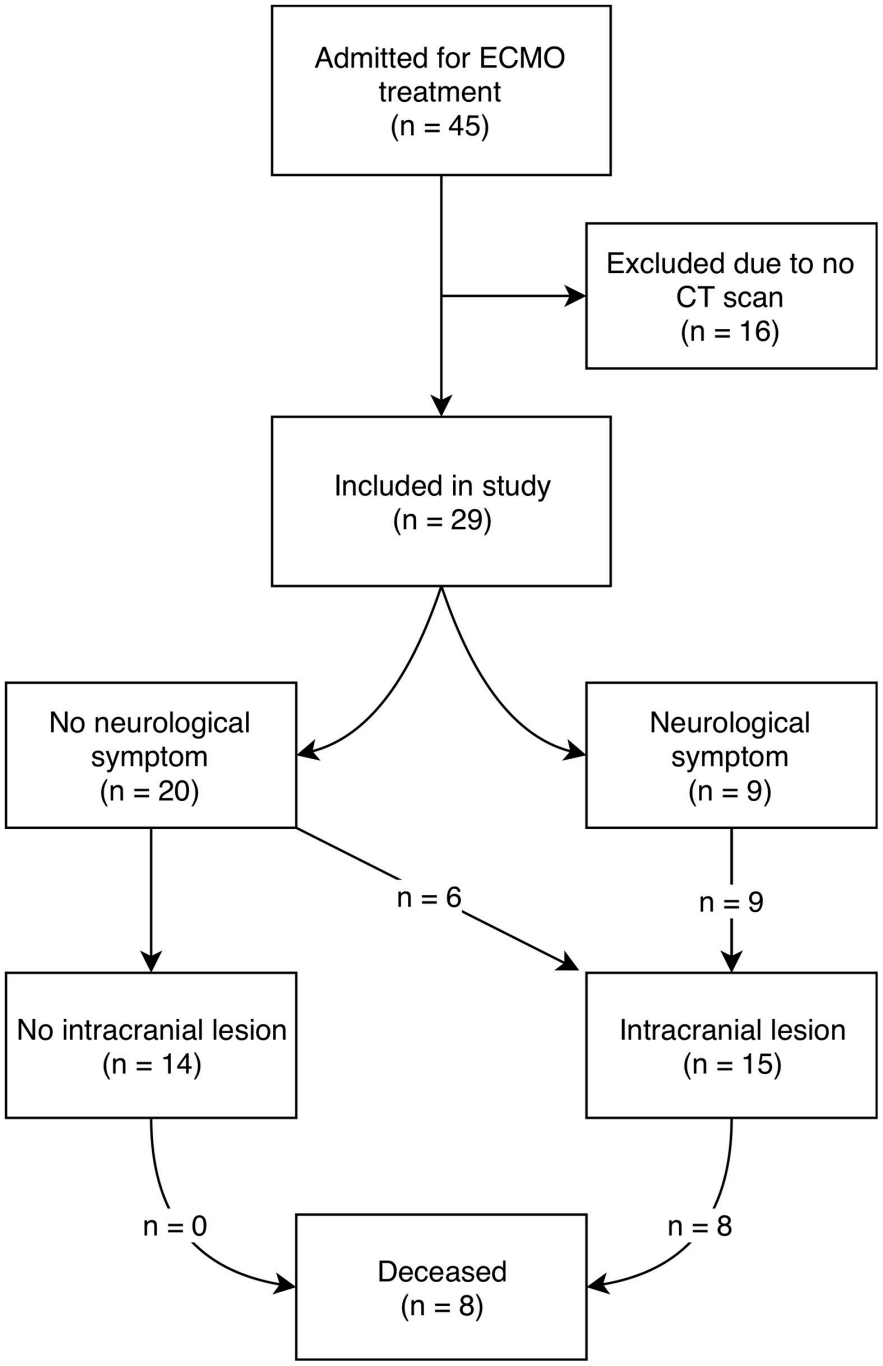
Schematic overview of patient inclusion and outcome. ECMO, extracorporeal membrane oxygenation; CT, computerized tomography.

During the study period, 45 patients were admitted for ECMO treatment. Of these, 29 patients underwent ≥1 CT scan(s) and were eligible for inclusion. Patient demographics for the included cohort are presented in Table 1. Patients were of varying ages, but the majority (41%, *n* = 12) were adults with a mean age of 50.4 years. The most frequent ECMO indication was infectious disorders, and the most common ECMO mode was venoarterial (VA). The median duration of ECMO treatment was 128 h (about 5 days). During ECMO treatment, 52% (*n* = 15) of patients suffered an intracranial lesion, however only 60% (*n* = 9) of these patients exhibited a neurological symptom preceding the diagnosis. In total, 28% (*n* = 8) of the included patients died during ECMO management, all of which had suffered an intracranial lesion and had higher S100B levels (Table 1) (Figure 1) (Supplementary Figure 4).

**Table 1:** Patient demographics.

**Variable**	**Entire cohort (*n* = 29)**	**Adult group (*n* = 12)**	**Pediatric group (*n* = 7)**	**Neonatal group (*n* = 10)**
Male sex	14 (48)	6 (50)	2 (29)	6 (60)
Age	N/A	50.4 ± 11	9.1 ± 5.3	N/A
Cardiopulmonary resuscitation preceding ECMO	8 (28)	1 (8)	3 (43)	4 (40)
VA ECMO	22 (76)	8 (67)	5 (71)	9 (90)
VV to VA conversion	3 (10)	1 (8)	1 (14)	1 (10)
VA to VV conversion	1 (3)	0 (0)	1 (14)	0 (0)
Length of ECMO treatment [hours (IQR)]	128 (76–336)	208 (94–417)	115 (80–461)	128 (84–150)
Pre-diagnostic symptom(s)	9 (31)	6 (50)	1 (14)	2 (20)
Intracranial lesion (any type)	15 (52)	7 (58)	4 (57)	4 (40)
Type of intracranial lesion	ICH only: 7 (24) Ischemia only: 5 (17) ICH and ischemia: 3 (10)	ICH only: 4 (33) Ischemia only: 1 (8) ICH and ischemia: 2 (17)	ICH only: 2 (29) Ischemia only: 2 (29) ICH and ischemia: 0 (0)	ICH only: 1 (10) Ischemia only: 2 (20) ICH and ischemia: 1 (10)
S100B (μg/L) (grand median)	0.49 (0.28–1.2)	0.80 (0.30–1.5)	0.42 (0.30–0.92)	0.40 (0.27–0.58)
Mortality	8 (28)	5 (42)	1 (14)	2 (20)

*Data is depicted as mean (SD) or median (IQR), if continuous. Categorical data is depicted as count (%). Of note, since the number of S100B measurements per patient varied, each individual does not contribute equal amount of data for the calculations of S100B. ECMO, extracorporeal membrane oxygenation; ICH, intracranial hemorrhage; VA, venoarterial; VV, venovenous*.

**Figure 2:**
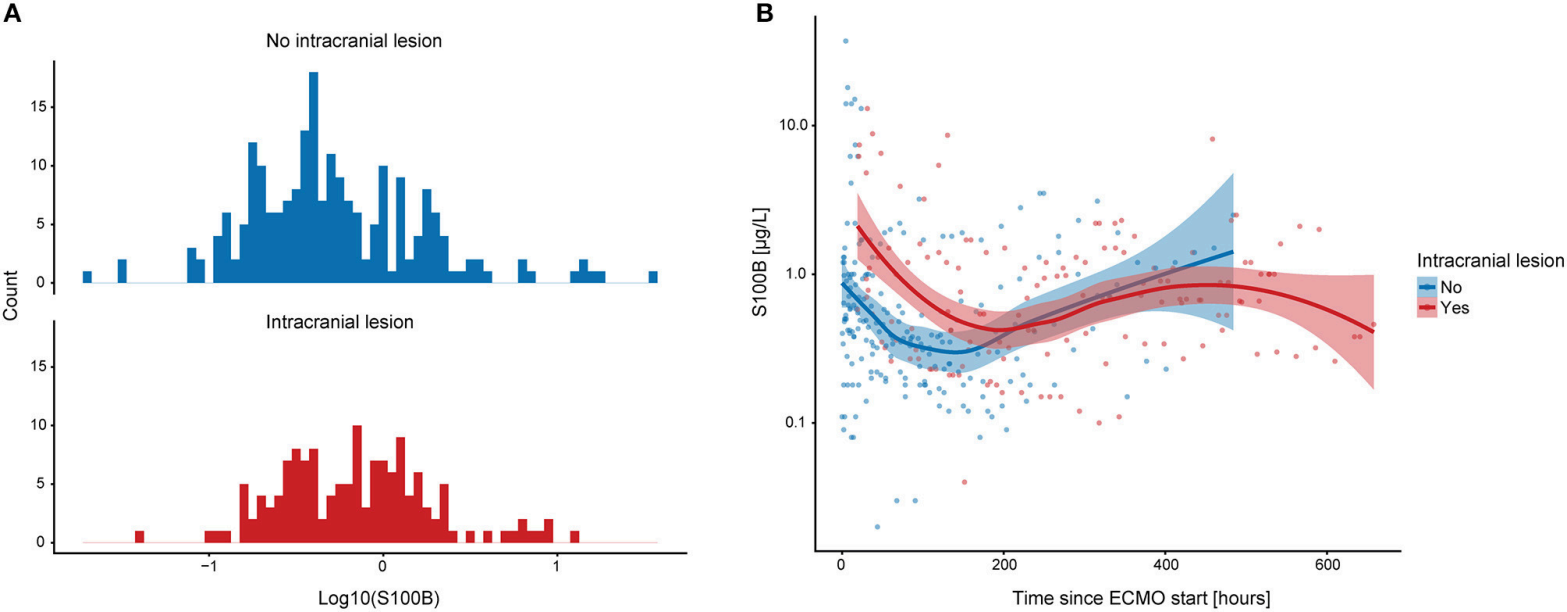
Graphical depiction of S100B in ECMO patients. The distribution of S100B values in ECMO patients subcategorized on intracranial lesion is shown **(A)**. Overall, ECMO patients that suffered an intracranial lesion seemed to have higher S100B values. In **(B)**, S100B is depicted longitudinally and subdivided similarly to **(A)**. The smoothened line indicates lowess curves and the shaded area surrounding it indicates confidence intervals. ECMO patients that suffer an intracranial lesion have higher S100B values during the first week on ECMO. ECMO, extracorporeal membrane oxygenation; lowess, locally weighted scatterplot smoother.

On a group level, patients diagnosed with an intracranial lesion exhibited slightly higher S100B values compared to patients without an intracranial lesion (Figure 2A). Portrayed longitudinally, S100B values were notably higher among patients who subsequently suffered a CT verifiable intracranial lesion during the first ~200 h following ECMO initiation (Figure 2B).

### S100B Predicts Intracranial Lesion Development After ECMO Initiation

**Figure 3:**
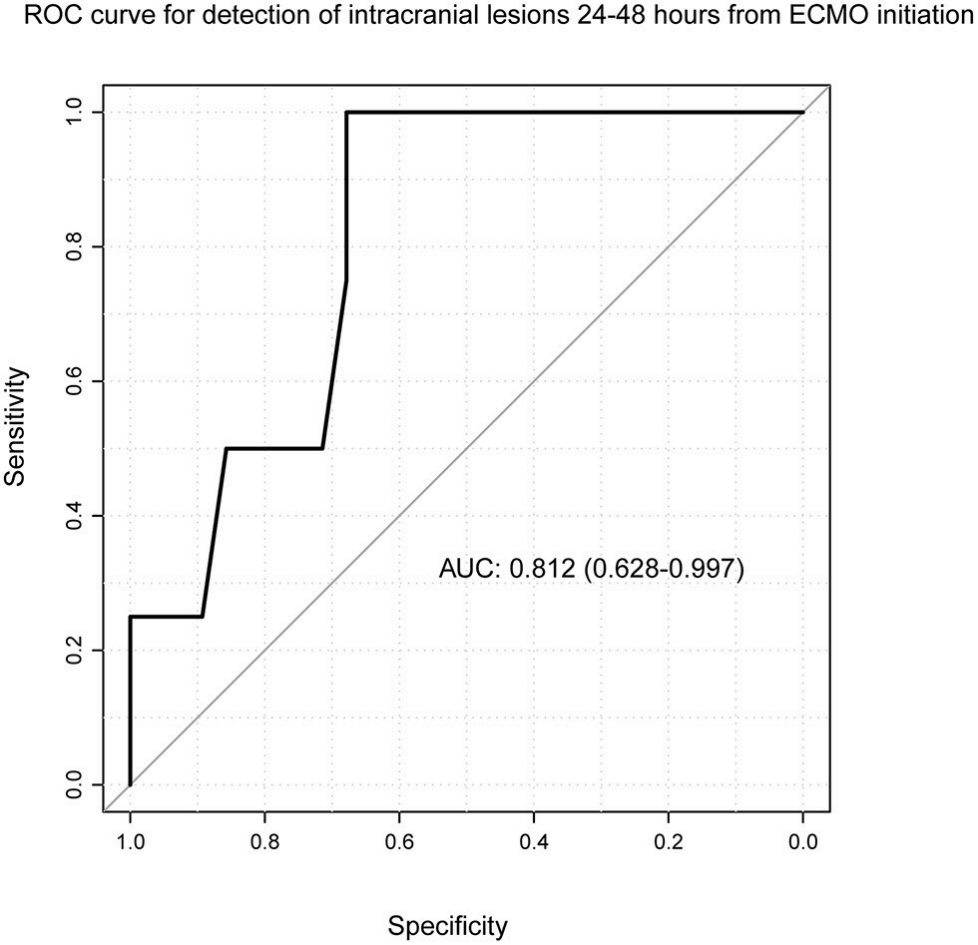
Receiver operating characteristics curve for S100B and intracranial lesions. Time since ECMO initiation was re-categorized into defined time intervals, during which one patient contributed one S100B value. One ROC curve was generated for each time interval, using intracranial lesion as dependent variable and S100B values during the time interval as independent variable. Here, the time interval of 24–48 h from ECMO initiation is depicted, demonstrating that S100B conferred an AUC value of 0.812 (CI: 0.628–0.997) meaning that S100B is a significant predictor of intracranial lesion among ECMO patients at this time point. Threshold analysis yielded a cut-off level for S100B of 0.69. ECMO, extracorporeal membrane oxygenation; CI, confidence interval; ROC, receiver operating characteristics.

A ROC curve was generated for each of the six pre-specified time intervals (excluding the fourth interval where no intracranial lesion was diagnosed), showing that S100B values obtained between 24 and 48 h after ECMO initiation conferred the best AUC (0.81, 0.628–0.997) to predict intracranial lesion development (Figure 3). Threshold analysis yielded a cut-off level for S100B of 0.69 μg/L. This threshold conferred a sensitivity of 100% (95% CI = 0.75–1.00) and a specificity of 68% (95% CI = 0.54–1.00).

**Figure 4:**
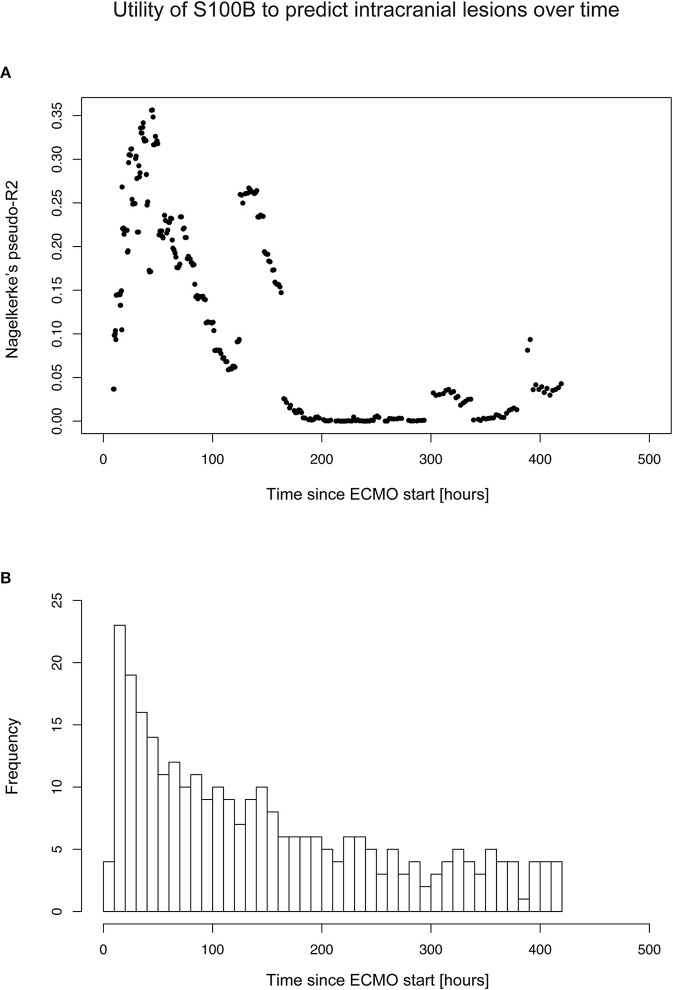
Timing of optimal S100B sampling to predict intracranial lesion development. Timing of optimal S100B sampling was determined using a sliding window approach, using a logistic regression approach with intracranial lesion as binary outcome and S100B as independent predictor. As shown in **(A)**, there were certain time points that conferred a high Nagelkerke's pseudo-R^2^, indicating that there might be time points of particular interest for S100B sampling. In **(B)** the distribution of S100B samples across the retrospective study population is shown, with the y-axis representing the number (*n*) of S100B samples within each time interval (x). IQR, interquartile range.

Using a sliding window approach, S100B conferred a Nagelkerke's pseudo-R2 of ≥ 30% during the second day of the ECMO treatment (median 37 h after ECMO initiation, IQR 30–46 h) (Figure 4A). This indicates that there are time windows of particular clinical relevance for S100B sampling. However, caution should be taken when interpreting this data, since the amounts of S100B samples was time-dependent (Figure 4B) and the number of observations were low due to the small number of included patients.

### S100B Increase Is Associated With all Types of Intracranial Lesions

Three different Cox proportional hazards models were calculated, using intracranial lesion and the respective subgroups (ICH and ischemia) as dependent variables (Table 2). In each model, S100B conferred a strongly significant and positive HR, meaning that S100B increments is indeed associated with a significantly higher risk for all type of intracranial lesions examined in the current study.

**Table 2:** Cox proportional hazards models for different types of intracranial lesions.

**Model #**	**Dependent variable**	**Independent variable**	**HR**	**HR, 95% CI**	***p* independent variable**	**Log rank test** **(robust) *p*-value**	**Wald score** ***p*-value**
1	Intracranial lesion	Log10-S100B	6.08	2.73–13.57	<0.001	0.007	<0.001
2	ICH	Log10-S100B	10.18	3.82–27.10	<0.001	0.02	<0.001
3	Ischemia	Log10-S100B	5.74	1.65–19.98	0.006	0.05	0.006

*Three different models comprising all patients but with different dependent variables are shown. In all analyses, log10-transformed S100B was the independent variable. Overall, all models were significant, which can be assessed using the Robust Log Rank Test and the Wald Score, which were used since they do not assume independence of clustered observations. S100B emanated as a significant predictor in Model #1–3. The interpretation of the HR in the case of a continuous variable, e.g., S100B, is that a one-unit increase is associated with a 6 times increased risk for any intracranial lesion, 10 times increased risk for ICH, and a 5.7 times increased risk for ischemic events. CI, confidence interval; ICH, intracranial hemorrhage; HR, hazard ratio*.

### A Secondary Increase of S100B Was Seen Following ICH Development

**Figure 5:**
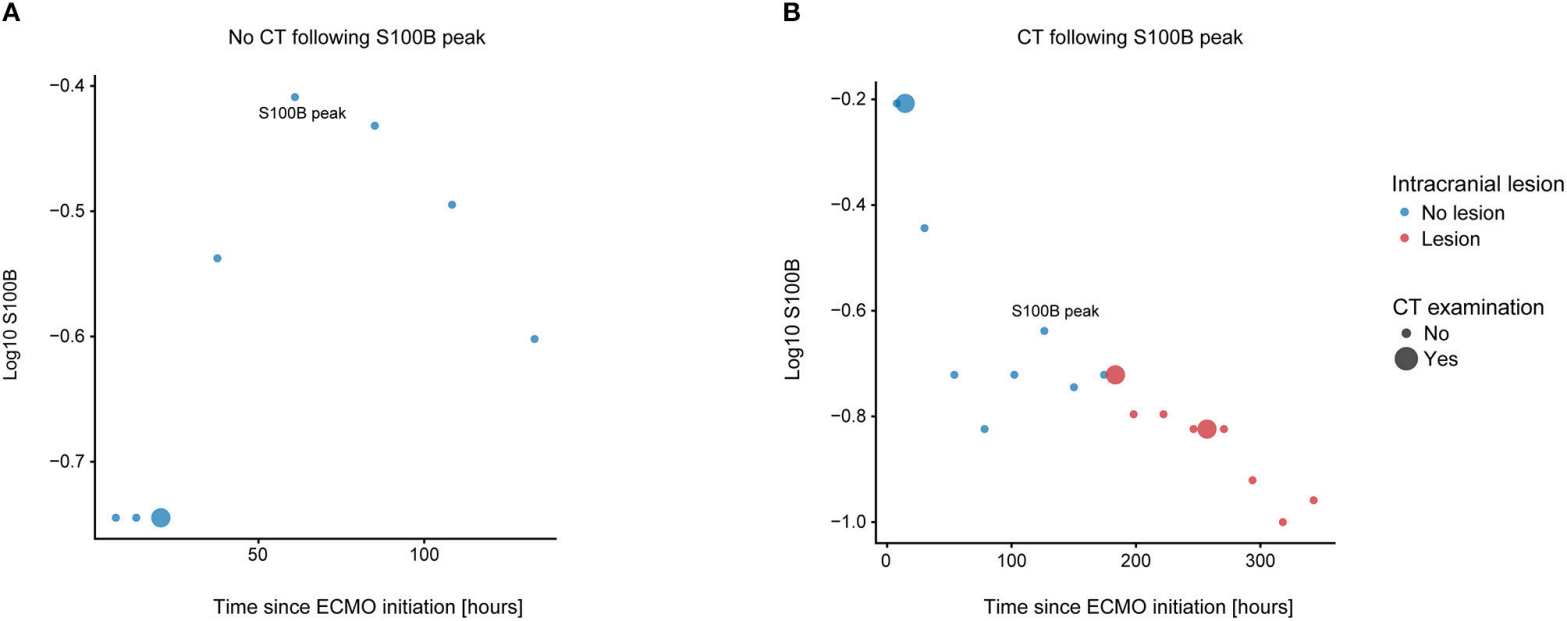
Subgroup analysis. We chose a subset of patients that had undergone a CT scan following a S100B peak and conducted a Cox proportional hazards analyses on these. We chose patients by eyeballing each patient's individual S100B trajectory, subcategorized by radiology examination and type of intracranial lesion. Patients that had not undergone any CT scan following a S100B peak were excluded, of which one representative patient is shown in **(A)**. **(B)** shows a patient that was included for subgroup analysis, since the patient had a secondary S100B peak and subsequently underwent a CT scan. CT, computerized tomography.

We performed a sub-group analysis of 23 patients that underwent a CT scan following a S100B-peak. A representative depiction of one excluded patient and one included patient can be seen in Figures 5A,B, respectively. Of note, Figure 5A also depicts a typical patient were the increase in S100B would have triggered a CT scan if it was used clinically. Similar to Table 2, three different Cox proportional hazards models were fitted to the subgroup data (Table 3). Results were similar with regard to intracranial lesion (HR 4, CI 1.8–9.0, *p* < 0.001) and ICH (HR 7 CI 2.5–19.6, *p* < 0.001) as dependent variables. For ischemia, the model failed to reach significance, making its results non-interpretable.

**Table 3:** Cox proportional hazards models in a subgroup analysis.

**Model #**	**Dependent variable**	**Independent variable**	**HR**	**HR, 95% CI**	***P* independent variable**	**Log rank test** **(robust) *p-*value**	**Wald score*p-*value**
1	Intracranial lesion	Log10-S100B	4.01	1.78–9.02	<0.001	0.03	<0.001
2	ICH	Log10-S100B	7.05	2.54–19.56	<0.001	0.03	<0.001
3	Ischemia	Log10-S100B	3.72	0.99–14.05	0.052	0.11	0.052

*Patients included in the subgroup analyses had all undergone a CT scan following a S100B peak. Similarly, to Table 2, three different models with different dependent variables are shown. In all analyses, log10-transformed S100B was the independent variable. Overall, Model #1–2 were significant, which was assessed using the Robust Log Rank Test and the Wald Score, since these do not assume independence of clustered observations. Since Model #3 was not significant, the results of this model are non-interpretable. S100B emanated as a significant predictor in Model #1–2. The interpretation of the HR in the case of a continuous variable, e.g., S100B, is that a one-unit increase was associated with a 4 times increased risk for any intracranial lesion, and a 7 times increased risk for ICH. CI, confidence interval; ICH, intracranial hemorrhage; HR, hazard ratio*.

## Discussion

Despite a limited sample size and diverse cohort, we found that S100B levels had a robust association with the development of both ICH and ischemic lesions during ECMO treatment, and that samples acquired between 40 and 140 h following ECMO initiation seemed to better predict lesion development compared to other sample times. Moreover, S100B was markedly increased following the development of an ICH. In summary, these findings highlight the potential benefits of serially sampled S100B to help detect intracranial lesion development during ECMO treatment.

We found that 52% of the included patients developed an intracranial lesion during ECMO treatment. While this is a high frequency compared to similar studies (7), we believe it is primarily due to our exclusion of ECMO patients that did not undergo a CT scan and institutional tradition of occasionally performing cerebral CT scans even in the absence of neurological symptoms (i.e., at the same time as a CT scan of the thorax or abdomen). Only 60% of patients that suffered an intracranial lesion exhibited neurological symptom(s), highlighting the difficulties involved in comprehensive clinical examination of ECMO patients. Mortality for the entire cohort was 28%, which is in accordance with current literature (32). All of the patients that died had developed an intracranial lesion, emphasizing the dire consequences of the complication.

On a group level, serum levels of S100B showed a robust association with the development of intracranial lesions. Samples acquired 40 and 140 h following ECMO initiation seemed to be better at predicting lesion development compared to other sample times, thus indicating a biphasic temporal pattern of S100B levels among ECMO patients. A tentative S100B threshold of 0.69 μg/L seemed to optimize sensitivity (100%) and provide an adequate specificity (68%) for lesion development detection during the second day following ECMO initiation. This cut-off should be interpreted with caution due to the small sample size and the choice of threshold calculation method (33). Of note, cut-off levels as high as 2.16 μg/L have been suggested for patients with severe TBI and unfavorable outcome (34). Thus, while 0.69 μg/L is high compared to healthy controls, it is lower than early/admission levels in severe TBI. This value is also higher than what would be expected in non-ECMO cohorts, for example neuro-critical care managed TBI patients serially sampled with S100B where a secondary increase of 0.10 μg/L, or even 0.05 μg/L, has yielded an adequate sensitivity/specificity for intracranial lesion development (17). Similarly high baseline levels have been shown in previous S100B sampled ECMO cohorts (35–37), and we believe this to be influence from an elevated serum “baseline” S100B level due to ECMO-associated extracranial sources of S100B (38). These could include heart failure, reperfusion injury, systemic hypoxia, acidosis, extracerebral injury, as well as the surgical aspects of cannulation, decannulation and performing a tracheostomy (39). Therefore, instead of determining an exact threshold, the combination of increased S100B levels and a rising trajectory might be best used as an indicator to perform a CT scan. As is shown in Figure 2B, the cohort that presented with intracranial lesion development presented higher S100B levels early on, perhaps as an indicator of pathophysiology foreboding deterioration. Moreover, patient subjected to pre-ECMO CPR had higher median initial S100B concentrations compared to the rest of the cohort [1.25 (0.73–16) vs. 0.5 (0.23–0.98)]. This is promising because if S100B is correctly implemented, it may highlight patients more susceptible to progressing CNS injuries already at admission.

Three studies have previously investigated S100B in ECMO populations. In a study of 15 patients, Nguyen et al. found that the three patients with cerebral complications had significantly higher levels of S100B at 5 days (120 h) following admission, possibly constrained by a limited number of patients making statistical modeling difficult. Interestingly, their mean S100B level of 0.799 μg/L in patients with cerebral complications supports our suggested threshold and that ECMO patients have an inherently higher S100B cut-off compared to non-ECMO cohorts (35). Another study of 80 pediatric ECMO patients found that S100B significantly predicted functional outcome (dichotomized Pediatric Cerebral Performance Category), but were not significantly higher in patients with CT verifiable lesions (36). However, they used grand medians of peak biomarker concentrations and did not look at trajectories, thus making comparisons to our own material difficult. While not proposing a cut-off for intracranial lesions, their 0.52 μg/L threshold for unfavorable outcome is also in the range of the cut off value for lesion development in our study. Lastly, Gazzolo et al studied S100B and TCD in eight ECMO treated infants, noting that S100B predated significant changes in pulsatility index in the middle cerebral artery (37). Thus, although restricted by small study populations, previous studies support the use of S100B monitoring in ECMO cohorts. Compared to these studies, our study benefited from a consecutive inclusion of all ECMO-treated patients during a predefined time period, exact time stamps for ECMO initiation/CT/S100B samples, unbiased S100B sampling, a larger sample size, and the employment of more robust statistical methods (such as ROC curves and Cox proportional hazards models).

In addition to S100B, other protein biomarkers of brain injury have been studied in smaller ECMO cohorts. In a study of 80 pediatric ECMO patients, Bembea et al found that peak concentrations of glial fibrillary acidic protein (GFAP) and intercellular adhesion molecule 5 (ICAM) were higher in patients with abnormal neuroimaging findings (36, 40). However, they used grand medians of peak biomarker concentrations and did not look at temporal dynamics. Another study of 65 extracorporeal cardiopulmonary resuscitation patients showed that higher serially sampled NSE values were associated with poorer neurologic outcome, but did not look at the relationship between NSE and CT findings (41). In our study, we did not include NSE as we believed that its longer effective serum half-life (14) would make it less effective in detecting cerebral lesions, as compared to S100B. Moreover, while a potentially additive effect can be attained by combining different biomarkers (42), S100B and NSE have not been shown to add any independent predictive value in TBI patients (14). Other previously analyzed proteins, such as ICAM5, brain-derived neurotrophic factor (BDNF), GFAP and chemokine (C-C motif) ligand 2 (CCL2) were not included as they do not have swift automated assays, thus making them difficult to apply in the clinical setting. In summary, we believe that S100B, despite its limitations, holds the greatest potential and clinical utility compared to other clinically available protein biomarkers but more studies are needed in order to establish independent utility if different proteins are combined.

## Limitations

This is a retrospective study, with its inherent limitations. For instance, 16 patients were excluded as they did not undergo a CT scan during treatment, which infers selection bias. Patients were also assumed not to have suffered any intracranial events preceding ECMO admittance. While this assumption is made in almost all ECMO studies of intracranial lesion development (8, 9, 43–46), recent evidence suggests that a substantial amount of ECMO patients (up to 15%) may have asymptomatic intracranial lesions prior to treatment (47). Furthermore, as clinicians were not blinded from the S100B result it is possible that S100B levels triggered a CT scan or altered patient management. However, we do not believe this to be a substantial limitation as our primary outcome was the development of intracranial lesions and not patient outcome *per se*. Because of the small sample size, we abstained from adjusting for different age groups, which is a limitation as S100B in healthy cohorts is higher in neonates (48). However, grand median S100B levels in the different age groups of our material revealed that adults had higher concentrations than both the pediatric and neonatal population (Table 1). Grand median S100B levels depending on age group and intracranial lesion development is also presented in the supplementary material (Supplementary Table 3). In the Cox model we also refrained from adjusting for any potentially important confounders, since we had a small study population and would have risked to overfit our data if adding more presumptive co-variates.

## Conclusion

Despite a relatively small and diverse cohort, serial serum S100B samples were both significantly elevated and had an increasing trajectory in ECMO patients that developed intracranial lesions. Prospective trials investigating S100B's clinical usefulness as part of an algorithm to detect neurological injury during ECMO treatment are warranted. Future studies should aim at prospectively collecting S100B, with a large homogenous patient cohort, while simultaneously conducting CT scans upon admission (baseline) and during pre-defined time-points to allow for un-biased S100B assessments.

## Data Availability

All datasets generated for this study are included in the manuscript and/or the Supplementary Files.

## Ethics Statement

Because this study did not modify existing diagnostic or therapeutic strategies, according to Swedish Law it was not necessary to obtain informed consent. The study was approved by the Regional Ethical Review Board in Stockholm, Sweden (#2018/830-31).

## Author Contributions

AF-S: data collection. CL and ET: statistical analysis. CL, ET, and AF-S: data interpretation. AF-S, CL, and ET: draft of manuscript. LB: study supervision. All authors: study design, revision and approval of manuscript.

### Conflict of Interest Statement

The authors declare that the research was conducted in the absence of any commercial or financial relationships that could be construed as a potential conflict of interest.

## Abbreviations

AUC: Area-under-curve
CT: Computed tomography
CI: Confidence interval
ECMO: Extracorporeal membrane oxygenation
HR: Hazard ratio
ICH: Intracranial hemorrhage
IQR: Interquartile range
NIRS: Near infrared spectroscopy
TBI: Traumatic brain injury
TCD: Transcranial Doppler
ROC: Receiver operating characteristics
SD: Standard deviation
VA: Venoarterial
VV: Venovenous.
