# Supplementary material for: Serial S100B Sampling Detects Intracranial Lesion Development in Patients on Extracorporeal Membrane Oxygenation
Source: Front Neurol. 2019 May 16;10:512. doi: 10.3389/fneur.2019.00512 (PMC6532588; doi:10.3389/fneur.2019.00512)
Supplement: Supplementary file 2 [file Data_Sheet_1.zip › Supplementary material.docx]

**Supplementary Figures and Figure legends**

**Supplementary Figure 1 (S1). Missing map of all variables in the data.**

Variables were plotted in order to visualize the extent of missing data in each variable and guide further data processing. The x axis depicts each variable of interest in the data and the extent of missing. The y axis depicts singular observations, which in this data denotes a time-point rather than a patient, since each patient had several measurements (long format data structure). As can be noted, there are many missing data points for the outcome variables. This is due to the fact that outcome was only assessed at the time points of a CT scan. Abbreviations: CNS, central nervous system; CPR, cardio-pulmonary resuscitation; CT, computerized tomography; ECMO, extracorporeal membrane oxygenation; ICH, intracranial hemorrhage.

**Supplementary Figure 2 (S2). Schoenfeld and Martingale residuals for Cox proportional hazard models (full data set).**

In the upper panel (A, C, E), the Schoenfeld residuals for Model #1-3 as presented in Table 2 are visualized. The output of the proportional hazard calculation (ρ and p-value) is presented in the graphs for the respective model. A p-value ≥ 0.05 indicated that the proportional hazard assumption was fulfilled. The dots represent the Schoenfeld residuals, while the filled line represents the smoothened curve and the dashed curves the confidence interval at two standard errors. In the lower panel (B, D, F), the corresponding Martingale residuals are depicted. The smoothened red line indicates the lowess approximation, which in all graphs (B, D, F) is centered around zero. Abbreviations: CNS, central nervous system; ECMO, extracorporeal membrane oxygenation; ICH, intracranial hemorrhage; lowess, locally weighted scatterplot smoother.

**Supplementary Figure 3 (S3). Schoenfeld and Martingale residuals for Cox proportional hazard models (subgroup analysis).**

In the upper panel (A, C), the Schoenfeld residuals for Model #1-3 as presented in Table 3 are depicted graphically. The output of the proportional hazard calculation (ρ and p-value) is presented in the graphs for the respective model. A p-value ≥ 0.05 indicated that the proportional hazard assumption was fulfilled. The dots represent the Schoenfeld residuals, the filled line the smoothened curve and the dashed curves the confidence interval at two standard errors. In the lower panel (B, D), the corresponding Martingale residuals are depicted. The smoothened red line indicates the lowess approximation, which in (B) and (D) is centered around zero. Abbreviations: CNS, central nervous system; ECMO, extracorporeal membrane oxygenation; ICH, intracranial hemorrhage; lowess, locally weighted scatterplot smoother.

**Supplementary Figure 4 (S4). S100B (all values) and mortality during ECMO.**

The distribution of S100B values among ECMO patients subcategorized by mortality during ECMO treatment is shown. Overall, ECMO patients that passed away during ECMO treatment seemed to have higher S100B values. Abbreviations: ECMO, extracorporeal membrane oxygenation.

**Supplementary Tables and Table Legends**

**Supplementary Table 1 (Table S1): Definitions of diagnosis categories**.

| **Original diagnosis** | **Recategorized definition** | **Number of patients** |
| --- | --- | --- |
| Acute lymphatic leukemia | Cancer | 1 |
| Diaphragmatic hernia | Neonatal disease | 1 |
| Extracorporeal cardiopulmonary resuscitation | Cardio-pulmonary disorder | 2 |
| Hydrops fetalis | Neonatal disease | 1 |
| Influenza | Infectious disorder | 6 |
| Meconium Aspiration Syndrome | Neonatal disease | 4 |
| Mitral valve insufficiency | Cardio-pulmonary disorder | 1 |
| Myocarditis | Cardio-pulmonary disorder | 1 |
| Pneumonia | Infectious disorder | 8 |
| Pulmonary hypertension | Cardio-pulmonary disorder | 2 |
| Sepsis, Septic shock | Infectious disorder | 2 |

The patients’ diagnosis that preceded ECMO treatment was recorded into organ-/disease specific categories. Abbreviations: ECMO, extracorporeal membrane oxygenation.

**Supplementary Table 2 (S2): Definitions of time intervals for ROC curve analysis**

| **Time interval [hours]** | **Count no CNS injury** | **Count CNS injury** |
| --- | --- | --- |
| [-0.0167, 24] | 64 | 3 |
| (24, 48] | 28 | 4 |
| (48,72] | 18 | 3 |
| (72,96] | 16 | 0 |
| (96, 120] | 15 | 1 |
| (120, 488] | 71 | 3 |

In order to generate one ROC curve per defined time-intervals, the time-points in the dataset were recoded into the intervals stated above. During the interval (72,96] hours, no patients suffered any CNS injury, preventing ROC curve analysis to be conducted for that time interval. Abbreviations: CNS, central nervous system; ROC, receiver-operating characteristics.

**Supplementary Table 3 (S3): Grand median S100B levels depending on age group and intracranial lesion development**

|  | **Intracranial lesion** | **No intracranial lesion** |
| --- | --- | --- |
| **Adult** | 0.99 (0.82-2.1) | 0.19 (0.14-1.0) |
| **Pediatric** | 0.39 (0.26-0.57) | 0.64 (0.36-1.22) |
| **Neonatal** | 3.2 (0.65-7.95) | 0.34 (0.28-0.45) |
